# The model of local axon homeostasis - explaining the role and regulation of microtubule bundles in axon maintenance and pathology

**DOI:** 10.1186/s13064-019-0134-0

**Published:** 2019-11-09

**Authors:** Ines Hahn, André Voelzmann, Yu-Ting Liew, Beatriz Costa-Gomes, Andreas Prokop

**Affiliations:** Manchester Academic Health Science Centre, Faculty of Biology, Medicine and Health, The University of Manchester, School of Biology, Manchester, UK

**Keywords:** *Drosophila*, neurodegeneration, axons, actin, cytoskeleton, microtubules

## Abstract

Axons are the slender, cable-like, up to meter-long projections of neurons that electrically wire our brains and bodies. In spite of their challenging morphology, they usually need to be maintained for an organism's lifetime. This makes them key lesion sites in pathological processes of ageing, injury and neurodegeneration. The morphology and physiology of axons crucially depends on the parallel bundles of microtubules (MTs), running all along to serve as their structural backbones and highways for life-sustaining cargo transport and organelle dynamics. Understanding how these bundles are formed and then maintained will provide important explanations for axon biology and pathology. Currently, much is known about MTs and the proteins that bind and regulate them, but very little about how these factors functionally integrate to regulate axon biology. As an attempt to bridge between molecular mechanisms and their cellular relevance, we explain here the model of local axon homeostasis, based on our own experiments in *Drosophila* and published data primarily from vertebrates/mammals as well as *C. elegans*. The model proposes that (1) the physical forces imposed by motor protein-driven transport and dynamics in the confined axonal space, are a life-sustaining necessity, but pose a strong bias for MT bundles to become disorganised. (2) To counterbalance this risk, MT-binding and -regulating proteins of different classes work together to maintain and protect MT bundles as necessary transport highways. Loss of balance between these two fundamental processes can explain the development of axonopathies, in particular those linking to MT-regulating proteins, motors and transport defects. With this perspective in mind, we hope that more researchers incorporate MTs into their work, thus enhancing our chances of deciphering the complex regulatory networks that underpin axon biology and pathology.

## Introduction

**Fig. 1:**
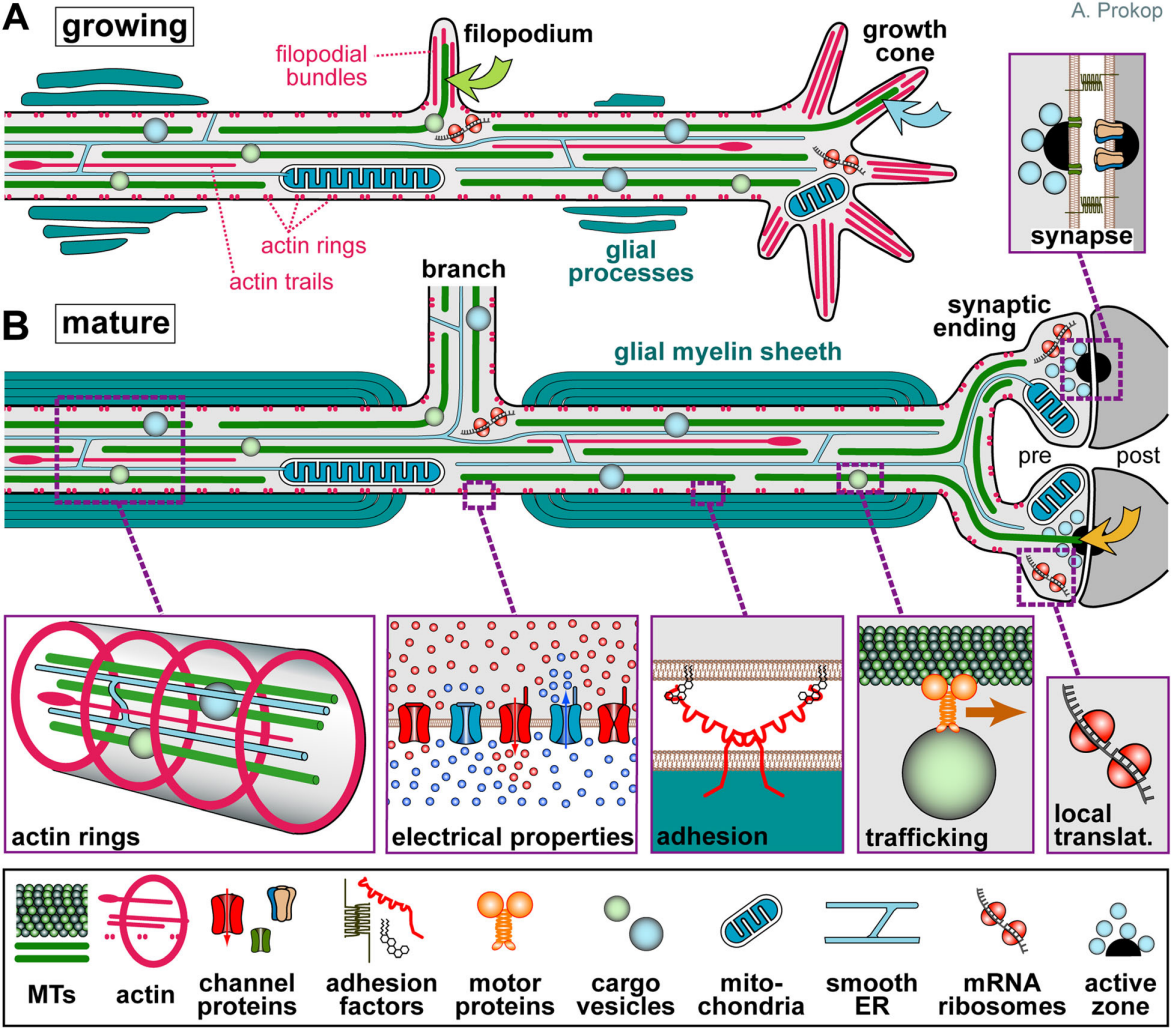
Specific properties of axons. Axons during the growth cone stage are shown in (**a**) and after synaptic maturation in (**b**), differing primarily in certain stage-specific specialisations including growth cones, synapses, electrical properties and glial interactions (here myelination [389, 392]). The core machinery in the axon shaft can be expected to be similar at both stages: parallel continuous bundles of extended but discontinuous MTs run all along axons serving as a structural backbone (see Fig. 2), a transport highway for axonal trafficking (driven by motor proteins), and a source for 'off-track' MTs contributing to morphogenetic processes including branch formation, directed axon growth and synapse formation/plasticity (green, orange, blue curved arrows); MT bundles are interspersed with longitudinal actin trails [18, 24], continuous networks of (smooth) endoplasmic reticulum [44, 393], and other membranous organelles including mitochondria [45]; axonal membranes display regularly spaced periodic rings of cortical actin [20, 21], a high number of ion-specific channel proteins and transporters to conduct nerve impulses [394], as well as adhesions with external structures including fasciculating parallel axons (not shown), glial processes [395] and synaptic partner cells [396]; a degree of independence from cell-body derived proteins is provided by local translation machinery [397–399] or supply from surrounding glia cells (not shown; [400–403]). Note that the axon diameter in the region between glia cells in B (referred to as Node of Ranvier) usually has a much smaller diameter than the rest of the axon [1]

Axons are the slender, cable-like extensions of nerve cells which form the nerves and nerve tracts that wire our brain and body, sending neuronal messages in highly regulated manners. With diameters of only 0.1-15μm [1], they extend over distances of up to a meter in humans. To adopt such a unique morphology and physiology, axons display many specialised features (Fig. 1).

Axons are indispensable for nervous system function, as illustrated by paralysis in spinal cord injury caused by the interruption of ascending and descending axon tracts [2, 3]. Axons are key lesion sites in injury-induced trauma and coma [4–7], and axon decay is believed to be an important trigger for neuronal decay in ageing and many neurodegenerative disorders [8, 9]. Notably, most neurons cannot be replaced, and compensation of lost axons through collateral branching of intact neighbouring axons has obvious limitations [9, 10].

This means that most axons have to be maintained for an organism's life time, i.e. up to a century in humans; unsurprisingly, mammals tend to lose almost half their axon mass towards high age [11, 12]. This trend is severely enhanced in neurodegenerative disorders, as illustrated by gradually increasing paralysis in spastic paraplegia or motorneuron disease [13, 14].

Research into neurodegenerative disorders typically approaches the problem by describing observed phenotypes and unravelling the molecular mechanisms performed by proteins linked to the disease. However, this approach rarely leads to satisfactory explanations of the pathology [15]. We believe that more profound understanding will arise when widening the scope from molecular to cellular mechanisms, by studying how proteins work within regulatory networks to underpin observable processes of axon biology - thus performing investigations at the same level of complexity at which pathology becomes manifest. Here we will illustrate this approach by focussing on the axonal cytoskeleton.

## The importance of microtubule bundles for axon biology

As illustrated in Fig. 1, the cytoskeleton of the axon shaft consists of straight parallel bundles of MTs, which are interspersed with intermediate filaments (not shown [16]) and longitudinal actin fibres called 'actin trails' [17, 18] - all running through a sleeve of cortical actin [19] which is now known to consist of evenly spaced periodic rings; these rings have been proposed to consist either of short and adducin-capped actin filaments [20, 21] or of two long intertwined actin filaments [22]. Significant deviations from this organisation, not to be considered in this review, exist at axon initial segments (not shown in Fig. 1), growth cones and synapses [23–26].

Of the three cytoskeleton classes, intermediate filaments were suggested by anatomical, developmental and genetic studies to regulate axon diameters, and their axonal aggregation is a hallmark of many neurodegenerative diseases [1, 27–30]. However, intermediate filament aggregations are not necessarily the cause, but can be the consequence of axon decay [30–32]. Notably, *Neurofilament-H-lacZ* mutant mice or *Quiver* mutant quail completely lack axonal intermediate filaments, but develop and breed fairly normally [33, 34]. Furthermore, various arthropods form axons of defined diameters in the absence of any axonal intermediate filaments [35–37]. In contrast to the moderate roles of intermediate filaments, actin and microtubules (MT) are essential for all stages of neuronal development and maintenance [37–39]. This review will be dedicated to the role and regulation of MTs.

**Fig. 2:**
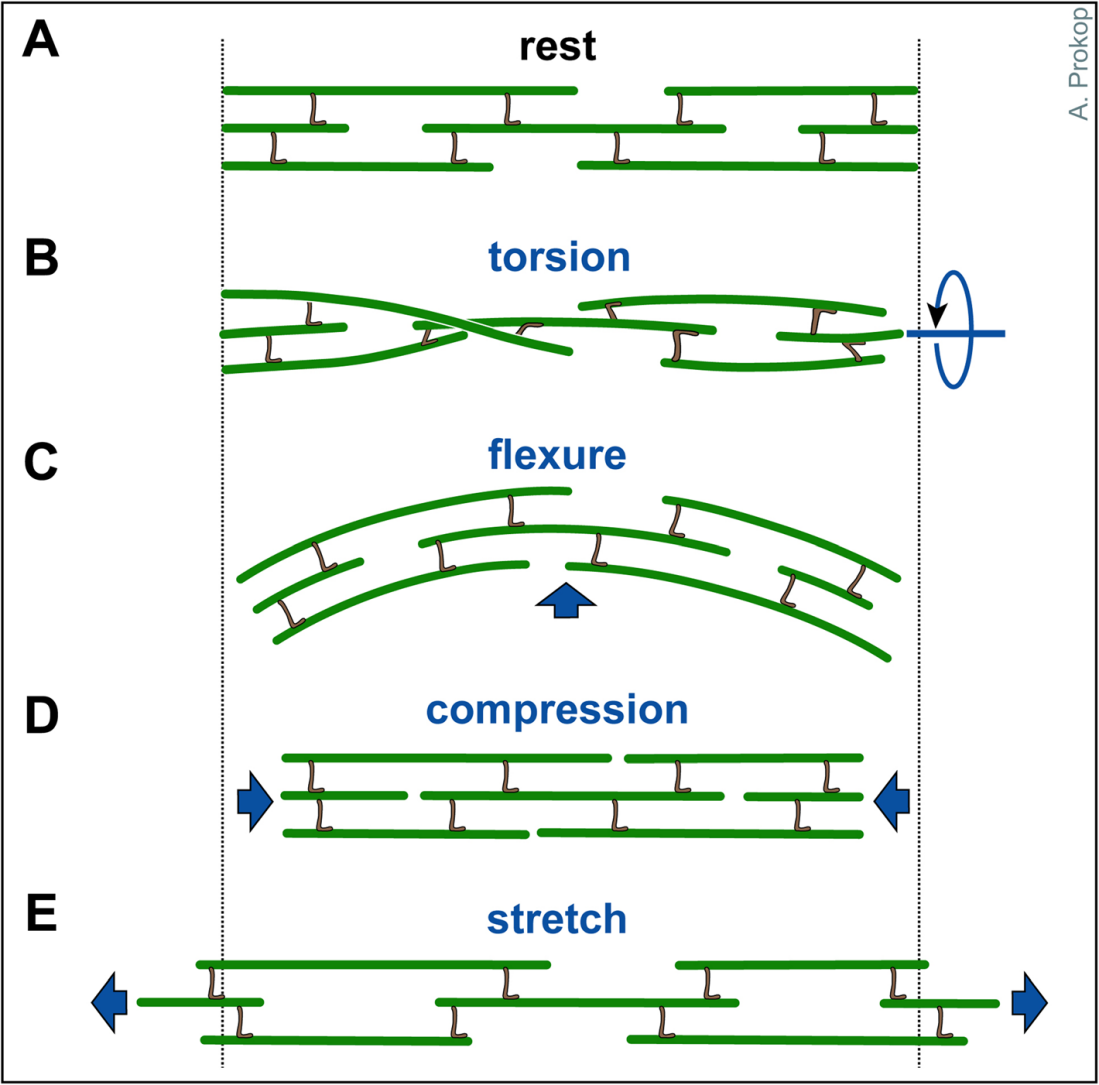
Axonal response to mechanical challenges. Continuous bundles of discontinuous MTs which are flexibly cross-linked (likely involving slip-bonds) are thought to provide a structural element that can respond to different forms of mechanical impact (as indicated in blue)

Axons contain bundles of MTs that run along the entire length of their shafts (Fig. 1); these bundles are essential for axon biology in at least three ways (details in Table 1): as structural backbones (Fig. 2), as highways for axonal transport and organelle dynamics, and as source for splaying MTs that can contribute to axon morphogenesis or physiology. Maintaining MT bundles is therefore crucial for axon longevity. Accordingly, there are prominent and numerous genetic links from MT regulators to hereditary neurodegenerative disorders (Suppl. Mat. in [46]), and axon decay is a frequent side effect of MT-targeting chemotherapies [53–56].

**Table 1:** Roles of axonal MT bundles

**Fig. 3:**
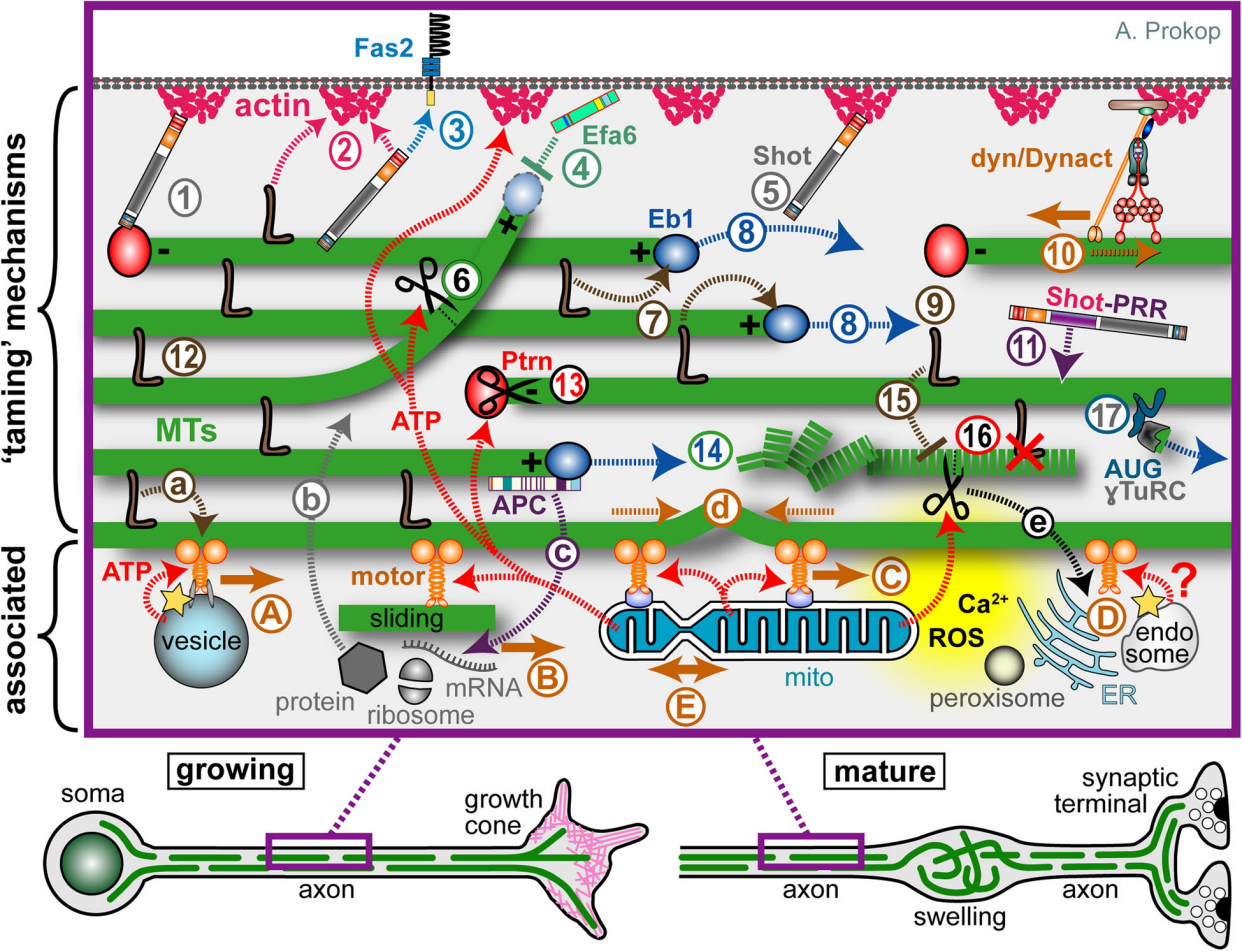
An interactome of MT-regulating and -associated mechanisms expected to contribute within the model of local axon homeostasis. Developing and mature neurons are shown at the bottom indicating that the close-up (magenta frame) might apply in both contexts. **1**-**16**) Potential mechanisms that can 'tame' MTs into bundled conformation: MT polymerisation (blue stippled arrows) is driven by molecular machinery centred on Eb1 (blue balls), further influenced by the tubulin supply machinery (not shown) and shaft-binding proteins (**7**); polymerisation generates new MTs required for bundle formation (**8**) and turn-over (**14**); to integrate into bundles, extending MTs require guidance via actin-Eb1 cross-linkage along the axonal surface (**5**; Shot) or along pre-existing MTs through MT-MT cross-linkers (**9**; brown L). The same or other cross-linkers provide the structural glue that holds MT bundles together (**12**; brown L); some of them can also bind to actin (**2**), they protect from (or recruit) MT severing activity (**15**), and influence motor protein dynamics (**a**). MTs which have escaped any cross-linkage are inhibited by cortical collapse factors when approaching the axonal surface (**4**; Efa6) or by MT-severing factors at MT-MT cross-points (**6**). The bundled MTs are discontinuous; their free minus ends are stabilised by CAMSAP/Patronin (Ptrn) together with katanin (black scissors; **13**), whereas non-polymerising MT plus ends are stabilised by other factors (not shown; e.g. CLASP or the Dynactin subunit p150/Glued [404, 405]). The dynein/Dynactin complex is believed to link cortical actin to MT bundles and drive them anterogradely (**10**), whereas Ptrn at minus ends may anchor MTs via spectraplakins to the axon cortex (**1**); spectraplakins may also link MTs directly to cortical actin (**2**) or to transmembrane receptors (**3**), and they are expected to perform further, still unexplored actin-independent bundle-promoting roles through their PRR domains (**11**). Tear-and-wear damages MTs (dashed green line), potentially affecting interaction with MT-binding proteins (**16**; red X); MT severing proteins might selectively eliminate such MTs (**16**; scissors), or MTs undergo repair (not shown). Nucleation of MTs (**17**) is mediated by ɣTuRC directionally anchored to MT lattices via the augmin/HAUS complex (AUG). **A**-**E**) Mechanisms closely 'associated' with MT bundles: MT-associated motor proteins ('motor', solid orange arrows) drive axonal transport of (protein-loaded) vesicles (**A**), cytoplasmic factors including proteins, translational machinery (ribosomes) or RNAs (**B**), move other MTs (**B**, sliding), and position/rearrange organelles including mitochondria (**C**, mitos), endoplasmic reticulum, peroxisomes and endosome (**D**) - and this likely includes mitochondrial fission and fusion (**E**). **a**-**e**) The motor-associated functions all act downstream of MT bundles because they require them to walk on; but they also act upstream: for example, the forces they generate (stippled orange arrows) are the potential cause for MT disorganisation (buckling shown in **d**); transport delivers important regulators and building blocks for bundle-maintaining processes (**b**); the proper regulation of organelles/endocytic compartments provides systemic factors that can orchestrate MT bundle-taming mechanisms, including intracellular free calcium or reactive oxygen species (Ca^2+^, ROS; yellow cloud [202, 203]) as well as ATP required for many processes including actin dynamics, MT severing and MT motor activity (red stippled arrows; note that vesicular transport uses glycolysis to generate its own ATP; yellow star); *vice versa*, the MT severer spastin also regulates the ER through ATP-independent mechanisms (**e**), and MT-associated proteins (APC) regulate local translation events (**c**)

*(1) Axonal MT bundles serve as structural backbones, not dissimilar to the vertebral column of a snake. Since MTs in these bundles are discontinuous and expected to be interlinked via flexible connections (see Section on cross-linkers), they are ideally suited to respond to longitudinal stretch and compression (similar to a half-extended telescope ladder), but also to torsion and flexure (Fig.* *2**).* *(2) Axonal MT bundles provide the highways for life-sustaining axonal transport between cell bodies and the axonal compartment. This transport is driven anterogradely by kinesins and retrogradely by the dynein/Dynactin complex; the cargoes include mRNAs, cytoplasmic proteins including signalling factors, vesicles delivering synaptic proteins, transmembrane proteins, neuropeptides and/or membrane lipids, as well as entire organelles including mitochondria (Fig.* *3**a-d* *[*40*–*44*]). Furthermore, local dynamics of organelles, such as fission or fusion of mitochondria, can be expected to require forces generated by MT-associated motor proteins (Fig.* *3**e* *[*45*]).* *(3) Axonal MT bundles provide a source for readily available MTs that can be used for other purposes (curved arrows in Fig.* *1**); for example, splaying MTs can trigger axon extension processes in growth cones* *[*26*,* 46*,* 47*], induce branching through growth cone splitting* *[*48*]* *or collateral branch formation along the axon shaft* *[*49*–*51*], as well as support physiological changes at synapses* *[*52*].*

**Fig. 4:**
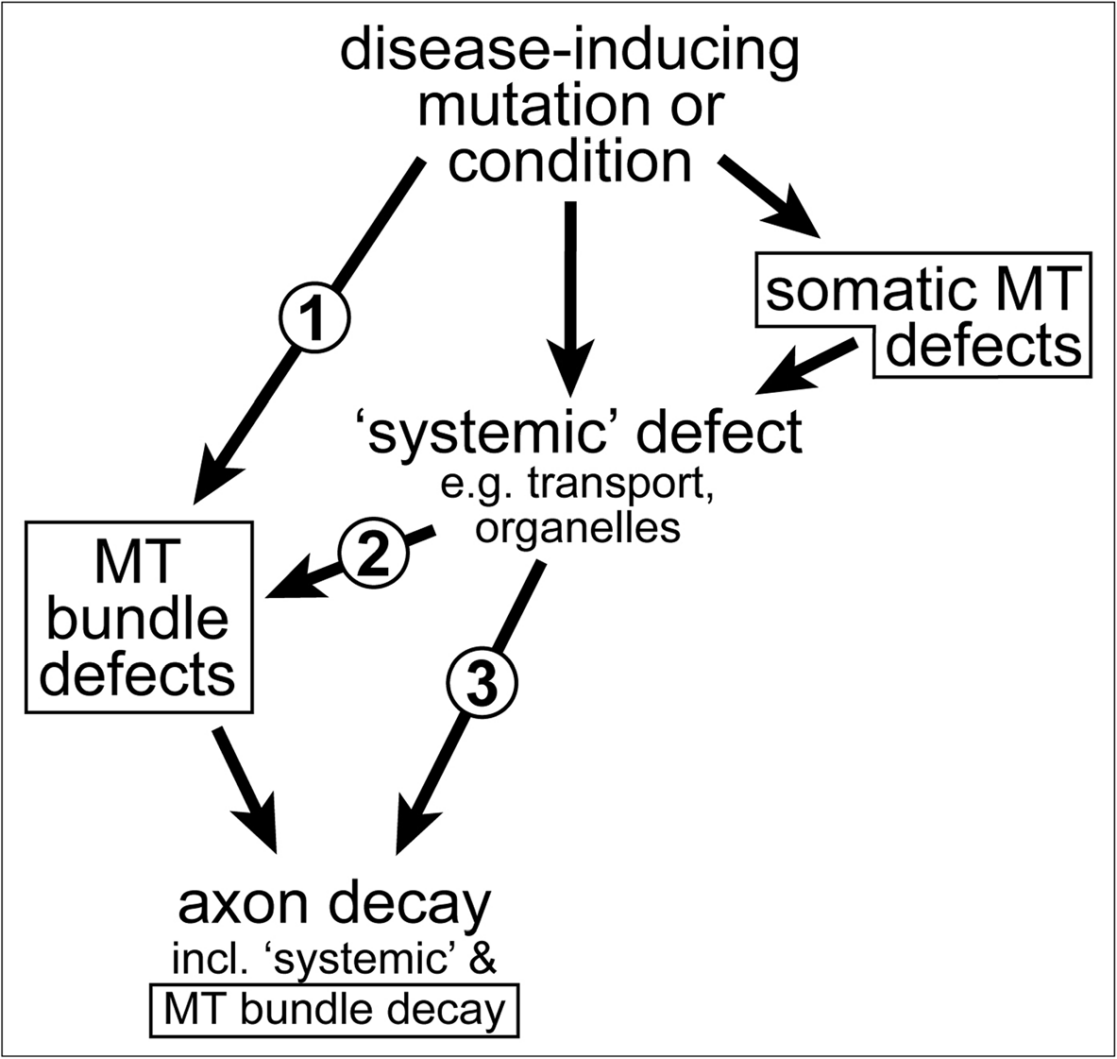
MT bundle defects as cause or consequence of axon decay. **1**) Disease-inducing mutations/conditions can affect a MT bundle regulator (e.g. dystonin [90]), thus causing MT bundle defects first which, in turn, can trigger axon decay. **2**) Disease-inducing mutations/conditions can affect systemic factors which, in turn cause MT bundle defects as an intermediate causative step in the cascade leading to axon decay (e.g. axonal transport fails, leading to MT bundle defects which then contribute to axon decay, as is the case in Alzheimer's disease or ALS [302, 406, 407]); this may occur even if MT regulators are affected, but these regulators mainly act in the cell body (e.g. dysregulation of the Golgi [408]). **3**) MT bundle deterioration may be a mere consequence of axon decay, although this case will be difficult to disentangle from option 2, since MT bundle disintegration and axonal disassembly may occur in parallel, as observed in developmental or injury-induced axon degeneration [409–411]). All MT-related phenotypes in this graph are indicated with a frame

Of particular interest for this review are reports of pathological axon swellings where MT bundles have disintegrated into loops or waves (bottom of Fig. 3), as observed in ageing, after injury and in certain *in vivo* models of axonopathies [7, 34, 57–66]. Notably, one study suggests that MT aberration upon ageing could cause swellings that trap and damage mitochondria, thus triggering axon degeneration [67]. However, MTs are surprisingly often ignored or side-lined in existing studies of axon pathology and there are simply not enough data to deduce meaningful correlations between axon degeneration and MT bundle decay. But even if this were to reveal a close correlation, this still would not exclude that, depending on the pathological condition, MT bundle deterioration may be a mere consequence rather than cause of axon decay (details in Fig. 4). Ultimate clarification will only arise from developing a better understanding of MT bundle-forming and -maintaining machinery. Here we propose a conceptual framework that may facilitate such developments.

## From work in *Drosophila* to the integrated model of local axon homeostasis

The foundations for this conceptual framework were laid when we took the decision to use the fruit fly *Drosophila melanogaster* as a means to study how cytoskeletal regulators collaborate in orchestrating the morphogenetic changes that drive axon growth [68]. *Drosophila* is certainly not a miniature human, but it has many advantages and provides powerful means to uncover the regulatory concepts behind the roles and regulations of axonal MTs, which then often apply to higher organisms (Table 2; [15, 69–71]). Through using *Drosophila* neurons as a consistent, standardised cell system, our group alone performed functional analyses of over 50 actin- and/or MT-binding or -associating regulators ([46]; unpublished data); these studies form an unprecedented pool of data on the basis of which to develop novel concepts [21, 72–76].

**Table 2:** Why use *Drosophila*?

*The use of Drosophila neurons to study the neuronal cytoskeleton has a number of advantages that were detailed elsewhere* *[*46*]. Key aspects are the high degree of evolutionary conservation of cytoskeletal proteins, regulators and dynamics, the experimental amenability of neurons in primary cell culture and in vivo* *[*46*,* 77*,* 78*], and the relative ease of genetic manipulation based on available resources and efficient combinatorial genetics* *[*79*]. The power of combinatorial genetics is rooted in the relative ease, speed and cost effectiveness with which genes can be manipulated and functionally analysed, facilitating also combined analyses of multiple factors in the same animals or cells* *[*46*,* 70*,* 80*]. Drosophila's combinatorial genetics has been extremely successful in overcoming problems of redundancy, and in generating new conceptual understanding of co-operative networks of neuronal MT regulation that underlie phenomena at the cellular level (see main text). This has similarly been demonstrated for C. elegans* *[*81*–*83*]. Such depth of understanding at the cellular level can hardly be achieved through isolated work on individual genetic factors.*	

**Fig. 5:**
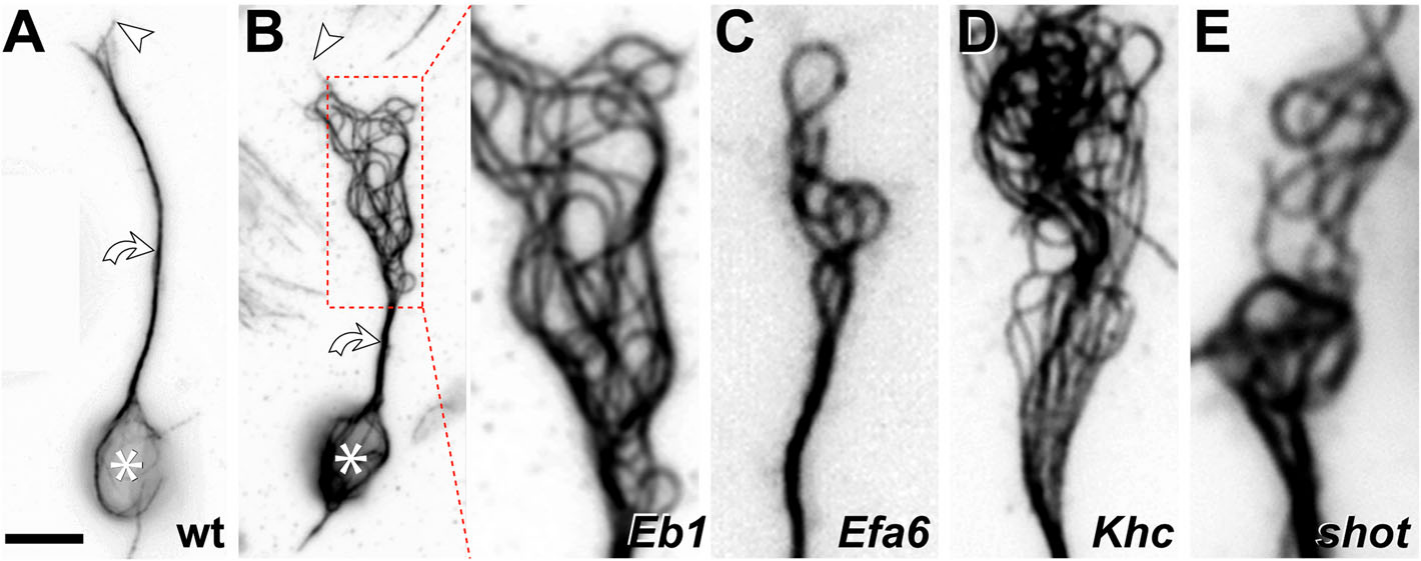
Disorganisation of axonal MTs upon loss of different MT regulators in *Drosophila* primary neurons. **a** Normal neuron (wild-type, wt) with soma (asterisk), axon shaft (curved arrow) and growth cone (tip of most distal MT indicated by arrow head). **b** *Eb1*^*5*^ mutant neuron where the area of MT disorganisation is framed by a red stippled box and shown as close-up on the right. **c-e** Similar close-ups shown for *Efa6*^*GX6[w-]*^, *Khc*^*27*^ and *shot*^*3*^ mutant neurons. Note that the four mutated factors perform fundamentally different molecular functions, with Eb1 being a MT plus-end binder ('8' in Fig. 3), Efa6 a cortical collapse factor ('4' in Fig. 3), Khc a kinesin-1 motor protein ('A-E' in Fig. 3) and Shot a multi-functional cross-linker ('1-3, 5, 11' in Fig. 3). All neurons were derived from wild-type or homozygous mutant embryos, mechanically and chemically dissociated, kept for 7 days in pre-culture in a centrifuge tube to deplete any maternal gene product, mechanically and chemically dissociated again, cultured on concanavalin A-coated glass coverslips for 1day at 21°C, fixed and stained with anti-α-tubulin (DM1A, Sigma; procedures detailed elsewhere: [78]); images were taken by A.V. using STED (stimulated emission depletion) microscopy. Scale bar in A represents 10 μm for the two neurons and 4 μm in close-ups

For example, loss-of-function conditions of 24 MT-binding or -associating (2^nd^ order) proteins that we analysed in cultured primary neurons, revealed MT disorganisation in more than half the cases. Interestingly, the MT disorganisation found in these various conditions appears to display certain common characteristics: axons display areas in which their bundles are dissolved into chaotic, intertwined, crisscrossing arrangements of curled MTs (see examples in Fig. 5). These phenotypes were surprising when considering that MTs usually behave like rigid rods [84–86]. Notably, when using some of the same genetic conditions *in vivo*, comparable phenotypes were observed in the fly brain [74]. Such *in vivo* phenotypes in the fly are reminiscent of the curled MT conformations in pathological axon swellings of mammalian models mentioned in the previous section. Potential evolutionary conservation of this phenomenon is further supported by the occurrence of similar MT curling and disorganisation in mouse and rat primary neurons [87, 88] - and we are certain that more reports will emerge once researchers consider MT disorganisation to be a phenotype worth quantifying.

As an attempt to explain the occurrence of this MT phenotype across mutant conditions and animal groups, we developed the model of '*local axon homeostasis*' [37, 89], based on two fundamental elements:

(1) The model proposes that MTs in axons show a strong bias to become disorganised and curl up. As detailed further below, this is most likely induced by the force-generating motor proteins that drive transport of large cargoes in the narrow axonal environment crowded with physical obstacles posed by organelles and protein complexes (Fig. 1, 'A-E' in Fig. 3). Once MT disorganisation occurs, e.g. through off-track polymerisation or buckling ('4' and 'd' in Fig. 3), it can form a seed that gradually develops into pathological axon swellings.

(2) The model further proposes that this risk is contained through the actions of different classes of MT-associating and -regulating proteins, which co-operate and complement each other to form robust machinery that 'tames' MTs into bundles ('1-17' in Fig. 3).

In this model, each axon segment uses locally acting MT regulators to maintain its MT bundles (hence 'local axon homeostasis'). Hereditary or acquired loss of single regulators would be expected to weaken this machinery and increase the statistical risk of MT disorganisation. Such heightened probability might explain why many axonopathies affect primarily long axons [54], and why certain disorders linked to MT regulators display late onset of axon decay [90].

In the next two sections, we discuss potential causes explaining the bias of axonal MTs to become disorganised. We will then summarise experimentally demonstrated MT bundle-maintaining mechanisms, and speculate about further mechanisms based on existing knowledge of known classes of axonal MT-regulating proteins.

## Understanding the unusual curling behaviours of MTs in axons

**Fig. 6:**
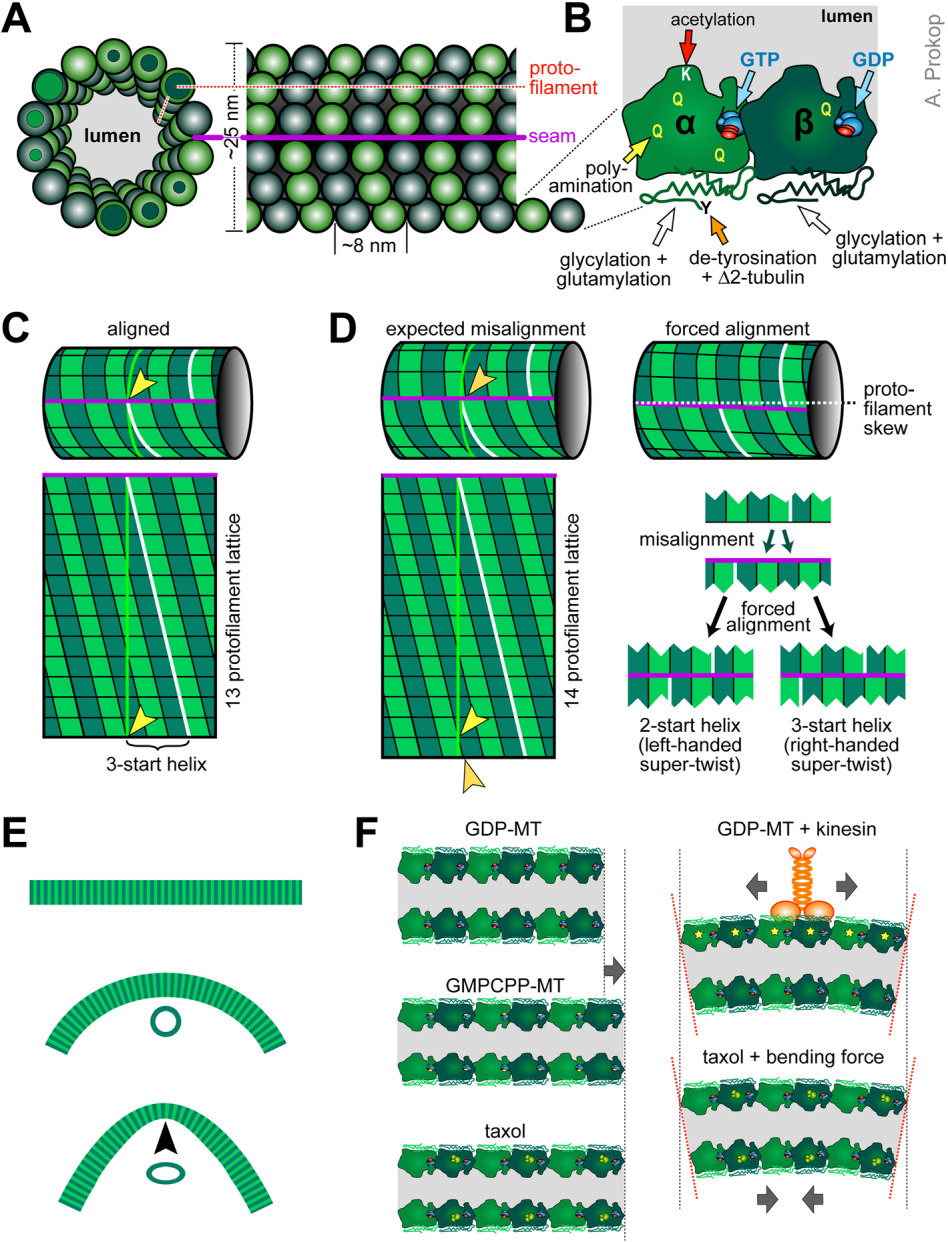
A molecular perspective of microtubule properties. **a** Cross-section of a MT with 14 protofilaments (PF) and lateral view of a 13 PF MT, both in B-lattice configuration, where α-tubulins make lateral bonds with α-tubulins and ß with ß, except at the seam (magenta line: seam; dashed red line: PF). **b** Close-up of an α/ß-tubulin heterodimer showing the various post-translational modification sites as indicated; note that the GTP of ß-tubulin in lattices is usually hydrolysed (GDP). **c** A 13 PF MT (top), cut open at the seam and rolled out (bottom); the yellow line shows the diameter, the white line follows the helical rise of laterally bonded tubulins; in 13 PF MTs, tubulins are precisely aligned at the seam (yellow arrow head) but shifted by three positions (3-start helix). **d** When deviating from the 13 PF prototype, tubulins are misaligned at the seam (orange arrow head); when forced into alignment, the PFs skew (deviation of the magenta line from the white stippled line), causing a super-twist of the MT as described by the 'lattice accommodation model' [98, 412]; for certain PF numbers, MTs can form two alternative alignments, of which usually the version with the lower helix start value (left) has a left-handed super-twist, whereas the higher value is right-handed [98]. **e** MTs behave like rigid rods with a persistence length of up to 10 mm, but can be bent down to diameters of curvature of ~1μm before they break; it has been reported that their cross-sectional profile may flatten above a certain threshold (black arrow head), thus softening the tube. **f** Lattices of GDP-tubulin are 1-3% shorter than MTs that were polymerised with the non-hydrolysable GTP analogue GMPCPP, or stabilised with taxol (orange structure binding α-tubulin in a 1:1 ratio, according to [413]); binding of kinesin-1 causes similar lengthening of tubulin (and additional compactions in the tubulin structure: yellow stars) which may cause cooperative binding of further kinesins and induce curvature if occurring only on one side of the MT; in extended taxol-bound MTs, bending forces were suggested to change tubulins on the concave side into their short conformation as an energetically favoured condition. For further references see main text

Although curvature is a key driver of MT plus end dynamics during de-/polymerisation [91, 92], MT lattices *in vitro* usually behave as rigid rods with a persistence length of 1-10 mm (as compared to ∼12 μm measured for actin filaments [84–86]). MTs are polar polymers composed of α/ß-tubulin heterodimers which are arranged in a head-to-tail fashion into linear protofilaments; usually 13 of these protofilaments are laterally aligned forming a straight tube of roughly 25 nm outer diameter (Fig. 6a, c). But MTs can deviate from this norm, and this may be one factor introducing an intrinsic bias towards disorder: for example, axonal MTs were reported to contain 13 protofilaments in frog olfactory or goldfish brain axons, but 11 or 15 in *C. elegans*, and 12 in certain neurons of *Drosophila*, crayfish and lobster [66, 93–95]. Deviation from the straight 13 protofilament conformation appears to equip MTs with functionally relevant physical properties [96, 97]. But it also introduces a skew into the MT structure, which causes a supertwist of the tubule (Fig. 6d [98–100]); this supertwist forces motor proteins to rotate around MTs [101] and is a potential explanation for supercoil of entire axons observed under destabilising conditions [83, 102].

Furthermore, MTs are structurally active: their physical properties can change when proteins bind to them (e.g. kinesins, see below) or when the 'tubulin code' is altered. The tubulin code is determined by the incorporation of different existing isotypes of α- and ß-tubulin into the MT lattice, and the addition of a range of distinct post-translational modifications (Fig. 6b [103–106]). Some modifications influence the interaction with MT-binding proteins (e.g. poly-glutamylation attracts spastin and tau [107, 108]). Others are believed to structurally protect MTs from damage or depolymerisation: for example, poly-aminations on various residues stabilises against depolymerisation [109], or acetylation of luminal lysine 40 makes MTs more flexible and break-resistant (Fig. 6b; [110–114]). Notably, site-directed mutation of lysine 40 in *Drosophila* α1-tubulin was able to demonstrate that intraluminal MT acetylation is physiologically relevant [115, 116]. In addition, the MT lumen may contain MIPs (MT inner proteins) that likely also modify MT stability [117].

These intrinsic or acquired physical properties are likely to determine how MTs respond to external forces - and we can expect such forces to be highly enriched in axons (see next section). Some ideas about how forces may impact on axonal MTs can be derived from *in vitro* experiments. For example, MTs in flow chambers that are anchored at one end, will bend when applying flow and rapidly return to straight confirmation when flow is stopped; if certain shaft-binding proteins (e.g. doublecortin or non-motile kinesin-1) are added, MTs become locked in bent conformation and fail to re-straighten [118–120].

Another example is provided by so-called *in vitro* gliding assays, where MTs are moved around on carpets of active motor proteins. On carpets of (axonemal) dynein, MTs move plus-end-first; they undergo collisions at high frequency, but seem to stay fairly straight and form vortices at the millimetre scale [121]. In contrast, if similarly prepared MTs are on kinesin carpets, they move minus-end-first and undergo fewer collisions because they can pass over one another, likely owed to the adaptable length and dynamic MT binding properties of kinesins [121–124]. However, if they collide or become pinned to the substrate (e.g. by dead kinesins) they can undergo dramatic shape changes including fishtailing and arc or loop formation at the micrometre scale [125–127]. The smallest diameters of curvature observed are similar to those of curled MTs in axons with values as low as 1-3 μm (Table 3, Fig. 5; [87, 88]) - and below 1μm MTs are believed to break [128, 129].

**Table 3:** MT loop or spool formation in gliding assays under different conditions. Footnotes: a) Primarily the lower range of mentioned diameters is listed; b) not clear from experimental section; c) measured from images. Abbreviations: CW, clockwise; CCW, counter-clockwise; polym, polymerisation; SA, streptavidin; tub, tubulin. References: (1) [126], (2) [134], (3) [138], (4) [137], (5) [130], (6) [135], (7) [136], (8) [414], (9) [133], (10) [132], (11) [415], (12) [121]. Note that a number of mathematical models were put forward to describe loop or spool dynamics in gliding assays [141, 156, 157, 414, 416, 417]

experimental conditions	diameters of curvature [μm] ^a^	comments	ref.
kinesin-1 carpets			
standard tub, 10-20 μm taxol (after?) ^b^ polym.	1-1.4 ^c^	waves and curls upon pinning	(1)
standard tub, 50 μM taxol during & after polym.; high MT density (2.5 MTs/μm^2^)	1-5	loops form through collision; loop duration frequently >5 min; strong increase in loops at high MT concentration; decreasing loop radius with increasing contour	(2)
rhodamine-tub, 10 μm taxol after polym.; exposing to air bubble or n-heptane	1.1 (heptane), 1.8 (air)	MTs become reversibly unstable in non-polar conditions: 50% of MTs form loops as long as close to air bubble; effect absolutely requires kinesins	(3)
rhodamine-tub, 10 μM taxol after polym.	2.5-3.75 ^c^	left-handed supertwist favours CCW rotation of loops; CCW rotation is preserved in spools	(4)
biotin-tub, 10 μm taxol after polym.; SA-linked	1-12.6, mean 3.9		
biotin-tub, 10 μm taxol after polym.; SA-linked	1-5, mean 2.3	up to 25 μm long straight bundles; pinning of tip induces spools or fishtailing; occasional “unspooling” events	(5)
biotin-tub, 10 μm taxol after polym.; SA-linked; 1600, 870, 270 and 90 kinesins/μm^2^	ca. 2.4-4	highest spool density & lowest spool diameter @ highest kinesin density; pinning as main cause for spool formation	(6)
biotin-GTP-tub, 10 μm taxol after polym.; SA-linked	5.7 (@ 10.8 μm length), 3 (@ 3,7 μm)	spool diameters increase with MT length per condition; spool diameters: GMP-MTs (taxol) < GMPCPP-MTs (no taxol) < GMPCPP-MTs (taxol)	(7)
biotin-GMPCPP-tub, 10 μm taxol after polym.; SA-linked	18.8 (@ 10.3 μm length), 5.8 (@ 3.4 μm)		
biotin-GMPCPP-tub, no taxol; SA-linked	8.2 (@ 10 μm length), 4.3 (@ 3.4 μm)		
biotin-GTP-tub, 10 μm taxol (after?)^b^ polym.; SA-linked	3.2 μm (@ 6μm length)	live imaging: pinning & collisions (simultaneous sticking) cause spool formation; spool formation is not activated by a Brownian ratchet type process	(8)
biotin-tub, 10 μm taxol after polym.; SA-linked; microfluidic device	2.7 (pinning), 6.2 (collisions)	live imaging: pinning & collisions (simultaneous sticking) cause spools of different diameters; pinning more frequent in flow cells than microfluidic device	(9)
biotin-tub, (taxol?)^b^ polym.; SA-quantum dot-linked	1.2, mean 3.4	left/right-handed super-twist: CCW/CW rotation; rings form intertwined wreath-like structures; tendency to disassemble involving MT breakage, kinesins pulling (blocked by AMP-PNP), counteracted by SA (enhanced by biotin)	(10)
biotin-tub, 10 μm taxol after polym.; SA-quantum dot-linked; patterned kinesin carpets	1-5.3 and 3.1	smallest spool diameters on constrained carpets: 1-5.3 μm on 5 μm stripes, 3.1 μm on 2 μm wide squares	(11)
axonemal dynein carpet			
Cy3-tub, 10μM taxol	straight	forming vortices in mm range	(12)

If MTs on kinesin carpets are reversibly cross-linked with biotin-streptavidin, they coalesce into bundles containing dozens of MTs which frequently curl up into spools with inner diameters similar to those of loops (details in Table 3). Spools can take on similar appearances as looped MT bundles observed in growth cones of fly or mammalian neurons [77, 130, 131]. Furthermore, single MTs can escape from spools which may trigger spool disassembly [130, 132, 133], bearing some resemblance with off-track MTs leaving axonal bundles ('4' in Fig. 3).

Parameters known to influence loops and spools *in vitro* might provide mechanistic insights into similar behaviours of MTs in axons (details in Table 3). Firstly, loop formation is favoured by high density of MTs and/or kinesins [134, 135], i.e. conditions that are clearly given in axons. Potential explanations are offered by reports that kinesins directly impact on the structural properties of MTs (see below), but they can also cause pinning events in gliding assays, which could be seen as a potential proxy for the abundant obstacles faced by extending or sliding MTs in the narrow axons. Secondly, higher MT rigidity results in larger diameter curls and spools in gliding assays [136]. Thirdly, right- versus left-handed supertwist of the MTs involved in gliding assays determines whether curls and spools have a clockwise or counter-clockwise directionality (Fig. 6d; [132, 137]). Furthermore, exposure to non-polar interfaces (e.g. n-heptane or air bubbles) induces strong curling [138], and this may be relevant in axons: changes in physical and chemical parameters of neurons upon ageing or in degenerative diseases promote liquid-liquid phase separation [139]; liquid compartments likely are of low polarity [140] and might therefore influence the curling bias of MTs. All these parameters observed *in vitro* can be expected to apply also in axons and might contribute to the observed curling behaviours (Fig. 5).

MT loops in gliding assays can be surprisingly stable (frequently >5 mins, as reported in [134]). To explain this, it has been proposed that tubulin-heterodimers on the concave side of the tube take on a shorter conformation than those on the convex side, and that this asymmetric distribution can be maintained as an energetically favoured state (Fig. 6f, bottom right [141]). Conformational length variations underlying this model were observed in non-hydrolysed GMPCPP-MTs where tubulins are 1-3% longer than hydrolysed GDP-tubulin; taxol added after (but not during) polymerisation achieves a similar elongation (Fig. 6f; [142–146]). Notably, this conformational length change seems physiologically relevant, as its suppression by the T238A mutation in yeast ß-tubulin stabilises MTs *in vivo* and causes mitotic defects [147, 148].

Such intrinsic properties of MTs may contribute to MT curling in axons, further influenced by MT lattice-associating proteins, such as tau, doublecortin or kinesin-1 which were reported to bind differently to curved *versus* straight MTs [118–120, 149–151]. In particular kinesins-1 was shown to extend MT lattices to similar degrees as taxol [120] through mechanisms that involve local compaction of tubulin different from taxol- or GMPCPP-induced effects [152, 153]. Since kinesin-1 has a preference for convex MT surfaces and was reported to undergo cooperative binding, this may lead to a curvature-enhancing and -stabilising snowball effect with an estimated diameter of curvature of 3.2 μm [120, 154, 155]. In this way, kinesin carpets in gliding assays might induce stable yet reversible curling, as has been suggested by mathematical modelling (top right in Fig. 6f, [156, 157]).

Naturally, current models are in their infancy and further findings need to be incorporated. For example, MTs behave as elastic cylinders (comparable to a garden hose) and can undergo softening through cross-sectional flattening when strongly bent (Fig. 6e [158, 159]). In this same vein, conformational changes of MTs upon kinesin-1 binding were reported to soften MTs locally [160]. If confirmed, this would have important implications for any existing models; together with the kinesin-induced tubulin compaction (yellow asterisks in Fig. 6f), it might be a mechanism to absorb energy and reduce the shear force load on MTs. Notably, softening of MTs is also observed upon taxol application (usually used in gliding assays; Table 3 [144]) or MT acetylation (abundant in axons [111, 114]), and might be a common prerequisite for curling behaviours.

To conclude, loop and spool formation in gliding assays are considered processes of 'active self-organisation' [125]. We strongly feel that this term might apply also to the formation of MT disorganisation in axons, and that potential mechanisms underlying MT curling in axons can be learned from *in vitro* assays. Notably, motor proteins, in particular kinesins, are being highlighted as key factors in both gliding and flow chamber assays. In the next section we will therefore summarise roles of kinesins during axon pathology.

## The intricate relationship between MTs and their associated motor proteins

Several kinesins display direct roles in MT regulation [161]: they may promote MT polymerisation (kinesin-2, -5 [162–164]), drive MT depolymerisation (kinesin-8, -13 [165]), stabilise MT-minus ends (kinesin-14 [166]), cross-link MTs (kinesin-5, -6, -12; see section on bundling), and regulate MT orientation as a feature of neuronal polarity [167–169].

However, in axons most attention is usually given to cargo and organelle transport/dynamics (Fig. 3a-e; see section on axonal cytoskeleton) driven retrogradely by the minus end-directed dynein/Dynactin complex, and anterogradely by plus-end directed kinesins (primarily kinesin-1, -2, and -3 [40, 170]). The forces imposed by these dynamics and/or the size of cargoes moved in the constrained environment of axons rich in physical obstacles, poses an obvious challenge to MT bundles [171] and might be an important factor leading to MT disorganisation.

Clearly, there is an intricate mutual regulatory relationship and finely tuned balance between the amount of transport and the structural properties of MT bundles as the transport highways [23, 171]. Thus, MT bundle properties influence transport: firstly, MT density is higher in small calibre axons than in large axons, with MT numbers and densities ranging to enormous degrees (4-130 MTs per axon, ~4-150 MTs/μm^2^); correlative studies and mathematical modelling suggest that higher MT numbers promote axonal transport ([172–174] and references therein). How MT numbers are so precisely controlled is an important but entirely unresolved issue that likely involves the nucleation machinery (see section on nucleation/polymerisation). Secondly, MT length correlates with transport rates [81]. Thirdly, the tubulin isotype composition of MTs, their posttranslational modifications, and the physical presence of certain MT-binding proteins influence motor protein dynamics ('a' in Fig. 3; [149, 175–180]).

*Vice versa*, transport affects MT bundles: for example, binding of kinesin changes the physical properties of MTs (see previous section), and binding and buckling through motor proteins cause damage to the MTs they walk on, triggering maintenance responses including MT repair or potentially even replacement ('14' in Fig. 3; [120, 181–185]). Close links between MT organisation and transport are also illustrated by charge-changing mutations in the H12 helix of *C. elegans* α-tubulin, reported to impact on axon transport whilst causing MT bundle aberrations [66].

Tipping the balance in this mutual relationship can easily be imagined to cause reciprocal deficiencies in transport rate and MT bundle organisation. For example, disorganisation or partial breakage of MTs has been reported to cause pathological transport deficits (option '1' in Fig. 4; [7, 67]). Furthermore, the space required for large cargo movements is likely generated through dynamic rearrangements of local MT-MT crosslinking networks (see section on cross-linkage); in this scenario, deviating from the right amount of cross-linkers may be a path to bundle aberration. *Vice versa*, demyelination upon immunological lesioning [186, 187], was reported to initially cause transport defects, which were then followed by MT disorganisation ('2' in Fig. 4; [60]). Analogously, we observe that loss of certain transport kinesins (Kinesin heavy chain/Khc/Kif5A or B, Unc-104/Kif1A) causes severe MT disorganisation in *Drosophila* primary neurons (Y.T.L. and A.V., unpublished data; Khc shown in Fig. 5e).

How loss of these kinesins may cause MT disorganisation can currently only be hypothesised. There are potential biomechanical and/or biochemical explanations. For example, it has been reported for dendrites that kinesin-1 migrates on acetylated and kinesin-3 on tyrosinated MTs [167]. Provided the same is true in axons, the loss of kinesin-1 would relieve acetylated MTs, but tyrosinated MTs would still bear their full transport load - and *vice versa*. Such imbalances in transport distribution within MT bundles could lead to shear forces that buckle MTs and seed MT disorganisation. Similar mechanisms may explain why MT disorganisation was observed at the axon initial segment upon directional changes in motor traffic caused by deficiency of the dynein regulator NDEL1 [188].

Loss of kinesins could have impact on MTs also through biochemical routes. For example, the bundle-maintaining machinery may simply suffer from aberrant supply of cargoes including (a) tubulin heterodimers as building blocks, (b) the MT-binding proteins that execute MT bundle maintenance work ('b' in Fig. 3), or (c) organelles which can be expected to play major roles in MT bundle maintenance (see Table 4 for details).

**Table 4:** The intricate relationship between MTs and axonal organelles

*MTs and cellular organelles display important and complex interdependencies. This becomes immediately apparent when considering that meaningful dynamics of any organelles will depend on MTs and their associated motor proteins (Fig. **3**C and D). Vice versa, organelles play crucial roles in cellular physiology directly or indirectly relevant for MTs, as outlined in the following for mitochondria:* *(1) Mitochondria are the main source for ATP [*195*], required to fuel multiple processes relevant for MT dynamics and regulation (red stippled arrows in Fig. **3**); these include actin assembly and dynamics [*196*,* 197*], phosphorylation of MT regulators [*198*], GTP production required for signalling events and MT polymerisation [*37*,* 199*,* 200*], MT severing [*201*], as well as MT-motor protein dynamics ([*40*]; but note that vesicular transport uses local glycolysis to generate its own ATP; [*202*,* 203*]*; *yellow star in Fig. **3* *A).* *(2) The mitochondrial surface is an important signalling platform potentially required to orchestrate MT regulation locally (not shown in Fig.* 3; *[*204*]).* *(3) Mitochondria cooperate with endoplasmic reticulum in the regulation of intracellular free calcium (yellow cloud in Fig.* *3*; *[*205*,* 206*]) which has direct impact on MT regulators (e.g. spectraplakins, tau, kinesins* *[*207*,* 208*]; or even on MTs themselves* *[*209*]).* *(4) Mitochondria collaborate with peroxisomes in the regulation of reactive oxygen species ('ROS' in Fig.* *3*; *[*210*,* 211*]), which have known effects on MT regulation* *[*212*]. If excessive amounts of the wrong ROS species are produced upon transport-induced mitochondrial damage or dysregulation of the mitochondria-peroxisome system, this causes oxidative stress as a major path to axon pathology* *[*67*,* 211*,* 213*]. Causative relationships between MTs and oxidative stress can be demonstrated experimentally: for example the MT-stabilising drug epothilone B rescues pathology caused by oxidative stress caused by peroxisome transport deficiencies in a human iPSC (induced pluripotent stem cell) model of SPG4 (spastin-linked spastic paraplegia 4*; *[*214*]), suggesting that MTs might be the cause for the transport deficit in the first place.* *Also other organelles impact on MTs. For example, the endoplasmic reticulum has multiple roles in lipidogenesis and protein synthesis but also calcium homeostasis* *[*44*], and the endo-lysosomal and proteasome-ubiquitination systems are required for proteostasis known to be relevant for MTs and axonal transport* *[*215*–*217*].*	

Functional interdependencies between transport and MT organisation provide potential explanations for a number of observations. For example, they may explain why axonal swellings induced by senile plaques in the *Tg-swAPP*^*Prp*^ mouse (overexpressing an amyloid precursor protein carrying a familial Alzheimer's disease-linked mutation [189]) were strongly enhanced when removing one copy of the KLC1 gene (kinesin light chain; a linker required for kinesin-1 mediated veciscular transport) - and this effect is conserved in *Drosophila* [190]. They may explain why different types of Charcot-Marie-Tooth disease or hereditary spastic paraplegias can be caused through motor proteins as well as regulators of membranous compartments [191, 192]. They may also explain why MT-stabilising drugs can be beneficial in animal models of neurodegeneration as diverse as SPG4 (Table 4) and Alzheimer's disease [193, 194].

Naturally, the argumentative framework presented here is highly speculative, given the enormous complexity of the relationships between MT bundle organisation, motor protein activity and organelle-dependent systemic factors. But we hope that these reflections will motivate experimenters to have a closer look at MTs in future studies of axon biology and pathology, and include statements in their reports as to whether MTs are affected. More data are urgently needed, which does often not require more than analysing neuronal morphology with antisera against tubulin (rather than restricting to intermediate filaments), or having a closer look at MTs in ultrastructural studies by increasing the resolution. In the following sections we will explore the mechanisms that are potentially used to form and maintain MT bundles against the odds of motor-induced aberration or damage.

## MT nucleation and polymerisation as fundamental requirements for bundle maintenance

The *de novo* formation of MT bundles during developmental, plastic or regenerative axon growth ('8' in Fig. 3) requires MT nucleation and polymerisation. Also in axons of mature and fully grown neurons, MTs undergo nucleation and polymerisation [37, 218], for example to drive MT repair and/or turn-over in order to maintain a steady state and prevent MT senescence ('14' in Fig. 1; [183, 184]). A well-regulated machinery of MT nucleation/polymerisation and disassembly is therefore needed to keep the numbers of healthy axonal MTs in balance with the transport load (see previous section; [172]).

Mechanisms of MT nucleation have long been known to be independent of centrosomes [219, 220] and should therefore involve cytoplasmic assembly or non-centrosomal MTOCs (MT organising centres; [221, 222]). For example, tau was reported to form condensations on MTs *in vitro* [179, 180], and such condensed phases of tau could theoretically have nucleation capacity [223, 224]. Furthermore, new MTs could arise from MT fragments (see Section on severing proteins below), potentially anchored via CAMSAP (calmodulin-regulated spectrin-associated protein)/Patronin to polymerise in the accurate direction towards the axon tip [113, 225]. Best demonstrated so far are mechanisms dependent on ɣTuRC (ɣ-tubulin ring complexes) and their anchorage via augmin/HAUS complexes to MTs ('17' in Fig. 3): depletion of either ɣ-tubulin or different HAUS proteins causes severe axon shortening and reduction in MT density; in addition, HAUS depletion causes polarity defects reflected in frequent MT polymerisation events towards the soma [226–228], suggesting that regulated nucleation is doubly important for axonal MT bundle maintenance. As the underlying mechanism it has been proposed that the augmin/HAUS complex anchors ɣTuRC to other MTs and points them distally ('17' in Fig. 3), and such a view is consistent with live imaging in *Drosophila* S2 cells [229].

The machinery of MT de-/polymerisation (blue stippled arrows in Fig. 3) requires at least three sub-machineries [37]: (1) dynamic protein complexes at the MT plus end that directly regulate polymerisation (blue balls, 'Eb1' in Fig. 3); (2) a complex regulatory network that supplies mature α/β-tubulin heterodimers as building blocks and that is closely co-regulated with MT dynamics ('B' in Fig. 3; [230–232]); (3) proteins binding or post-translationally modifying MT lattices that have impact on plus end dynamics, for example by stabilising MTs against depolymerisation or by promoting rescue ('7' in 'Fig. 3).

The fine-tuning of the net rates of MT nucleation and polymerisation appears to depend on complex regulation. For example, we recently found that loss of cortical actin rings in the axon shaft of *Drosophila* primary neurons (Fig. 1) caused a reduction in MT polymerisation speed, eventually affecting MT bundle integrity; simultaneous genetic or pharmacological destabilisation of MTs exacerbated these effects, frequently even eliminating entire axons [21]. Similar dependencies of MT polymerisation on actin networks are suggested by other reports: (1) parallel loss of spectrin and tau causes a reduction in axonal MT numbers in *C. elegans* [83]; (2) axon-shortening induced by the MT-stabiliser taxol can be ameliorated through co-application of actin-destabilising drugs (in both chick and *Drosophila* neurons [77, 233]); (3) application of actin-destabilising drugs changes the tubulin-to-microtubule ratio in PC12 cells [234] and causes axon retraction in chick dorsal root ganglia neurons ([235]; see also Table 5). Explanations for the mechanistic links from actin networks to net MT polymerisation remain speculative: they might involve biochemical pathways since cortical actin rings have recently been reported to act as signalling hubs [236], or might work through biomechanical mechanisms (see Table 5).

**Table 5:** Biomechanical models of axon growth

*The net rate of axonal growth has been proposed to be regulated through a balance between MT- and actin-dependent forces* *[*47*,* 240*,* 241*]. In axons, “actin is under tension supported in part by microtubules under compression” [*234*,* 242*]. Tension is provided by the pull of the growth cone [*243*–*245*] and the active contraction of acto-myosin, potentially the actin rings in the axon shaft (Fig.* 1*; [*241*,* 246*]; the stiff nature of cross-linked MT bundles is well suited to oppose compressive forces up to a certain threshold ([*240*,* 247*]; Fig.* 2*).* *In such a balanced system, manipulations such as externally imposed pulling forces* *[*248*–*251*]* *or genetic/pharmacological destabilisation of acto-myosin* *[*234*,* 235*,* 252*–*255*]* *clearly modulate axon length or growth. Part of this response is expected to be due to changes in MT assembly, as was found when applying external forces to non-neuronal cells* *[*256*]. MTs are not only responders in this context, but can generate forces themselves through dis-/assembly or motor-based sliding* *[*91*,* 252*,* 257*,* 258*].* *How forces are sensed and translated into compensatory force generation and/or changes in axonal length or growth, remains an important question (see also the last section on cortical anchorage). Potential mechano-responsive mechanisms might involve conformational changes of MTs (single MTs polymerise faster when being pulled in vitro) or changes in the activity status of polymerases such as XMap215* *[*91*,* 259*]. Furthermore, good experimental support exists for roles of mechano-sensitive calcium channels in axon growth control* *[*260*–*262*]* *and it remains to be seen whether this occurs through changing MT assembly/disassembly processes.*	

## Maintaining MT bundles through cortical guidance and elimination of polymerising MTs

Whilst MT nucleation and polymerisation are essential for axon formation and maintenance, they also pose a risk: for example, extending MTs may be obstructed by the abundant organelles or protein complexes in axons, thus causing accidental 'off-track' MTs that project out of the bundle towards the cortex ('4' in Fig. 3). Apart from MT buckling, off-track MTs may therefore be a second cause for axonal MT disorganisation.

A key factor preventing this from happening is Eb1 (end binding protein 1; Figs. 3 and 5b; [75]). Eb1 directly binds at extending MT plus ends where it promotes polymerisation [237] and serves as a scaffold for many other proteins [238]. Upon absence of Eb1 in *Drosophila* primary neurons, MTs are severely disorganised, indicating important roles in MT bundle maintenance (Fig. 5B [75]). One underlying mechanism is the guidance of polymerising MTs through binding of Eb1 to Short stop (Shot); Shot is a well-conserved spectraplakin, able to cross-link cortical actin, MTs and Eb1 ('5' in Fig. 3), thus guiding polymerising MTs in parallel to the axonal surface and laying them out into parallel bundles [75]. Accordingly, also loss of Shot causes severe MT disorganisation in fly axons (Fig. 5e) - and the same is true for functional loss of its two mammalian homologues ACF7 and dystonin in culture and *in vivo* [57, 59, 87, 90]. Of these, dystonin mutations link to the axonopathy HSAN6 (type 6 hereditary sensory and autonomic neuropathy [239]).

Cortical guidance is complemented by at least one quality control mechanism [74]: MTs that have (accidentally) left their bundled arrangements and extend towards the cortex in *Drosophila* neurons, get inhibited by Efa6 (exchange factor for ARF6; '4' in Fig. 3). Efa6 is a cortical collapse factor that associates with the axonal membrane via its C-terminal plekstrin homology domain and blocks MT polymerisation via its N-terminal MTED (MT elimination domain); when Efa6 is absent, off-track MTs outside axonal MT bundles persist for longer and are higher in number. Consistent with the known roles of off-track MTs in axon growth, branching and MT disorganisation (see Table 1 and above), fly neurons in culture and *in vivo* lacking Efa6 display longer axons, more branches and prominent MT disorganisation (Fig. 5d [74]).

Our model would predict that mutant phenotypes caused by loss of Shot and Efa6 should enhance each other because they are caused through complementary mechanisms of MT bundle regulation. Accordingly, we found enhanced MT disorganisation when removing both Shot and Efa6, and over-expression of Shot could rescue *Efa6* mutant phenotypes [74]. We propose therefore that Shot and Eb1 keep MTs away from the membrane, whereas Efa6 acts as a quality control factor inhibiting occasional accidental off-track MTs. This elimination seems to occur in moderate, well-balanced amounts so that 'intended' off-track MTs required for axon growth and branching can persist and perform their function.

Interestingly, the cortical collapse function of fly Efa6 is not conserved in vertebrates [74]. Nevertheless, the concepts derived from Efa6 studies appear relevant, because loss of the unrelated neuronal cortical collapse factor KIF21A (kinesin family member 21A; a type 4 kinesin) causes analogous phenotypes in mammalian neurons. Thus, KIF21A mutations linked to the neurodevelopmental disorder CFEOM1 (type 1 congenital fibrosis of the extraocular muscles) affect axon growth and axonal branching just like Efa6 [74, 263] - and might as well cause MT disorganisation, but no data are currently available.

However, guidance along cortical actin seems not the only mechanism through which Eb1 and Shot keep MTs on track. This is illustrated by the simple fact that MT disorganisation observed upon loss of Shot or Eb1 in primary fly neurons (Fig. 5b, e) does not occur when removing actin from axon shafts [21, 75, 77]. This suggests that both factors perform additional, actin-independent functions or interactions to promote MT bundles.

For example, the unusual Shot-PH isoform, which is highly enriched in the nervous system and harbours a plakin repeat region (PRR; conserved in mammalian spectraplakins), is a likely candidate for such roles that still await investigation ('11' in Fig. 3; [79, 90]). Apart from spectraplakins, Eb proteins have a long list of further interactors [238], and some of them might associate with MTs and guide extending plus ends along pre-existing bundles ('9' in Fig. 3); for example, APC (adenomatous polyposis coli) or GAS2-LIKE family members are good candidates, known to bind both MTs and Eb1 in mammals and *Drosophila* [264–266]. In this context, Eb1-APC-kinesin complexes were already suggested to contribute to MT guidance [169, 267]. Furthermore, MT guidance through septins might offer new explanations for axonal growth defects observed upon septin deficiencies [268–270].

## Potential roles of severing proteins and MT-destabilising kinesins in MT bundle maintenance

Apart from cortical MT elimination, also MT severing and/or depolymerisation in the cytoplasm may play important roles in maintaining axonal MT bundles. This is supported by axonal MT disorganisation observed upon the losses of *Drosophila* katanin (Y.T.L., unpublished results) or mammalian spastin [62–65].

As explained in the previous section, MTs leaving the bundled conformation can drive axonal growth, branching and MT disorganisation, and cortical collapse factors negatively regulate all three processes. In line with this argumentation, also Kif2A (a MT-depolymerising kinesin-13 family member; [271]) and MT severing proteins (spastin, katanin and fidgetin) were reported to inhibit neurite growth and/or branching [272–274]. However, other studies of spastin, katanin and fidgetin led to contradictory findings, describing them as promoters rather than inhibitors of neurite growth and branching [63, 275–282]. Such stark, potentially context-dependent deviations reflect the complex regulation of these proteins.

Spastin, katanin and fidgetin are all members of the superfamily of AAA proteins (ATPases associated with diverse cellular activities; [201, 283, 284]), but their severing activity is differentially regulated through their individual responses to (a) posttranslational MT modifications (in particular acetylation and poly-glutamylation; [107, 274, 285–288]), (b) competition with other MT shaft-binding proteins such as tau ('15' in Fig. 3; [275, 289, 290]), or (c) spatial recruitment through specifically localised proteins such as CAMSAP ('13' in Fig. 3 [291]). Furthermore, katanin has the ability to depolymerise MTs in an ATP-independent manner [292].

Through this context-dependent spatiotemporal regulation of their activities, severing proteins can have two diametrically opposed outcomes: they either eliminate MTs, or they break them up into stable fragments that may serve as seeds for MT amplification [113, 201, 293]. In the following, we will briefly speculate how either of these outcomes could be used to prevent MT disorganisation:

First, MT severing proteins could complement roles of cortical collapse factors ('4' in Fig. 3) by serving as quality control factors that eliminate disorganised MTs within the cytoplasm ('6' in Fig. 3). For example, katanin in plant cells was reported to localise and sever preferentially at MT cross-points, which can be used to take out non-aligned MTs [201].

Second, MT shortening functions of katanin are required at MT minus ends. Thus, in both mammals and *Drosophila*, the minus-end capper CAMSAP/Patronin protects against MT disassembly, and recruits katanin to counterbalance against uncontrolled minus-end polymerisation ('13' in Fig. 3; [225, 291, 294]); uncontrolled minus end extension upon katanin deficiency may cause MTs to go off-track or potentially buckle through extra forces produced. Note that CAMSAP3 also plays roles in maintaining non-acetylated MTs, thus further complicating interpretations [295].

Third, MT elimination functions could prevent MT bundle senescence. For example, MTs suffer from damage through tear-and-wear [120, 181–183, 185], which might cause bundle aberration by abrogating interactions with MT-binding proteins (red cross at '16' in Fig. 3). MT fractures or holes can be repaired through mechanisms involving katanin or spastin [183, 232, 293, 296, 297]. However, more subtle features of senescence (e.g. irreversible modifications, loss of tubulin C-tails) might require selective elimination of ageing MTs through severing factors (as similarly suggested for kinesin-8 or -13 [298]) which could then trigger compensatory polymerisation ('14' in Fig. 3). For example, spastin deficiency in the *Sp*^*Δ*^ mouse model caused a drop in MT polymerisation (potentially reflecting reduced turn-over) accompanied by a rise in MT disorganisation (potentially caused by precocious MT senescence; [65]).

However, the MT phenotypes observed in the *Sp*^*Δ*^ mouse model could likewise be explained through the opposite role of spastin in promoting MT multiplication through generating nucleation seeds: in the absence of such a function, MT numbers might gradually decline and cause transport interruptions and eventually axonal pathology (see section on motor proteins; [214, 299]). Curiously, axon swellings in the *Sp*^*Δ*^ mouse model were reduced with low doses of MT-stabilising or -destabilising drugs [65], thus failing to provide any clues as to whether spastin works through MT turn-over or amplification in this context.

Understanding spastin is important because it is by far the most prominent factor linking to spastic paraplegias worldwide [300, 301], and axonal swellings are a hallmark of the disease [192, 302]. Most SPG4-linked mutations lie within the AAA-ATPase domain [303], suggesting that MT severing is key to the disease pathology. However, point mutations might generate versions of spastin, which either act as dominant negative alleles (forming dysfunctional complexes that titrate out other spastin-interacting factors), or acquire gain-of-function qualities by diffusing away to perform very different roles. One such MT-independent role of spastin is the isoform-specific regulation of the endoplasmic reticulum ('e' in Fig. 3), including its shape, its interaction with the endosome and its production of lipid droplets [304–307]. It is therefore difficult to exclude that at least part of those SPG4-linked mutations triggers axon decay through other routes than the direct induction of MT aberrations ('2' or '3' versus '1' in Fig. 4).

## Potential roles of MT-MT cross-linkage in MT bundle maintenance

MT-MT cross-linkage appears an obvious means of suppressing MT disorganisation ('12' in Fig. 3) and is likely the oldest mechanistic concept put forward by neurobiologists to explain MT bundles [16, 308, 309]. Mathematical models support MT-MT cross-linkage as an important structural feature of axons (e.g. [310–313]), and physical cross-linking strands of varying length between axonal MTs were observed decades ago [16, 314]. Such MT-MT cross-linkers would be expected to detach upon super-threshold pull or compression, and re-attach thereafter (Fig. 2; slip-bonds), giving axons properties approximating those of active fluids (K. Miller, personal communication). However, the molecular players mediating MT-MT cross-linkage in axons remain surprisingly controversial to this day [315], as briefly explained in the following.

First, showing that a neuronal linker expressed in non-neuronal cells induces MT bundling, is insufficient proof: MT bundling can even be achieved through expression of isolated MT-binding domains, or the application of the MT-stabilising drug taxol; intriguingly, taxol-induced bundles display ultrastructural cross-bridges that are indistinguishable from those induced by tau or MAP 2 [308, 316–319]. As a further example, dynamin is linked to Charcot-Marie-Tooth disease and has been shown to bundle MTs *in vitro*; however, the physiological relevance of this is questionable, because dynamin *in vivo* seems to bind primarily membranes [320–322].

Second, neurofilaments were reported to fill spaces between MTs especially in larger diameter axons and to form lateral extension that link to MTs around them [323]. However, as mentioned in the second section, lack of neurofilaments is not a lethal condition, suggesting that cross-linking roles of intermediate filaments are not crucial in axons.

Third, MAP 1B appears an ideal cross-linker at first sight, because it possesses an N- and a C-terminal MT-binding domain, but it is not a convincing bundler when expressed in non-neuronal cells [324] - although the *Drosophila* homologue Futsch was reported to promote MT spools at synaptic terminals [325]. We are aware of only two reports mentioning axonal bundle defects upon loss of MAP 1B or Futsch [326, 327] and another where loss of Futsch reduces the spacing between axonal MTs [328]. Rather than MT bundle structure, most insights into MAP 1B/Futsch functions concern axon development, which likely reflects its major role [324, 329–331]. Another candidate with N- and C-terminal MT-binding domains is MTCL1 (microtubule cross-linking factor 1) which displays prominent bundling activity when expressed in non-neuronal cells and is prominently expressed at the AIS (axon initial segment) of cerebellar Purkinje cells [317, 332, 333]. Ultrastructural analyses of a range of neuron types have revealed that MTs at AISs are not distributed throughout the axonal lumen as observed along axon shafts, but are grouped up into parallel sheet-like arrangements cross-linked by ~25nm long spacers ('Axon' chapter in [334]). In Purkinje cells, these parallel arrangements are affected upon loss of MTCL1, as is in agreement with its proposed role as cross-linker [332]. However, due to its limited expression in the cerebellum, MTCL1 cannot explain similar sheet-like arrangements in the AISs of other neuron types. A more likely candidate is TRIM-46 (tripartite motif-containing protein 46) which is expressed in AISs in many nervous tissues, contains only one central MT-binding domain, can induce sheet-like arrangements when expressed in non-neuronal cells, localises to the cross-bridges, and its knock-down in cultured neurons causes reduced cross-linkage [335, 336].

Fifth, the conserved linker candidate tau, has one central MT-binding region and seems to achieve physical MT-MT linkage through N-terminal dimerisation [337–339]. However, its dwell time on MTs is very short [150, 340]. Similar to MAP 1B/Futsch, we are aware of only rare reports of fairly mild bundle aberration upon loss of tau [83, 341], and most tau-deficient phenotypes concern neuro-developmental defects instead [331].

Pinpointing roles of tau or MAP 1B/Futsch in axonal MT-MT cross-linkage is enormously complicated by the fact that both proteins seem to perform a whole array of further molecular functions relevant for MT dynamics. For example, tau can protect MTs from severing by katanin [289], bind tubulin hetero-dimers [342], switch between bundled and single MT states [343], cross-link MTs with actin or the cortex [344–347], stabilise MTs [315, 348], maintain labile domains along MT shafts [290, 349], regulate end-binding proteins [350], compete with kinesins or regulate their traffic [179, 180, 351], and promote MT nucleation and polymerisation [331, 352]. A similarly broad functional pleiotropy has been reported for MAP 1B [324].

Gaining experimental proof for MT-MT cross-linking activities in axons is also complicated by functional redundancies. For example, enhanced phenotypes are observed when mutations of *MAP1B* and *tau* or of spectraplakins and *tau* are combined in the same neurons, or when Futsch and Tau are co-expressed. Such functional redundancies likely extend to further potential cross-linkers. For example, kinesin-5 (KIF11), kinesin-6 (KIF23, Pavarotti in *Drosophila*) and kinesin-12 (KIF15) slide anti-parallel MTs in the mitotic spindle [354]; since axonal MTs are arranged in parallel, these kinesins seem therefore to inhibit sliding in this cellular compartment [355–360], suggesting that they cross-link MTs. In support of this idea, we observe that loss of Pavarotti causes axonal MT disorganisation in *Drosophila* primary neurons which might reflect potential linker function (Y.T.L., unpublished data).

In conclusion, MT-MT cross-linkage is a long proposed concept, but pinpointing the responsible molecular factors in axons remains a key challenge. We seem to have come a step closer at the AIS, but are far from understanding the situation in the axon shaft. We even cannot fully exclude a model where MT bundles are held together by the corset of contractile cortical actin rings (Fig. 1), and cross-linkers merely separate MTs to generate space for transport [241, 309]. It is therefore pivotal to decipher the true molecular nature of MT-MT spacing/cross-linkage in axons; overcoming functional redundancies between different classes of linker candidates might be an important strategy to this end.

## Does MT bundle maintenance involve their anchorage to the axonal surface?

Apart from cross-linking MTs within axonal bundles, they might also be anchored to the axon wall, as a further means to prevent MT buckling and bundle deformation caused by the enormous forces imposed by axonal cargo transport. For example, axolinin in squid giant axon has been discussed as a potential MT-cortex linker [361]. Certainly, large ankyrin isoforms can anchor MTs to the cortex at the AIS of mammalian neurons [25, 362, 363] and along axon shafts in *Drosophila* [328]. In the case of *Drosophila* it was proposed that MT-cortex linkage through ankyrin combined with spacer function of the MAP 1B homologue Futsch (which contains an unusual central domain of 60 repeats with neurofilament homology [329]) form networks that sustain a large axon diameter, thus replacing roles of neurofilaments in mammals (see above).

Relevant in this context is the phenomenon of slow (ca. 0.5-5mm/day) somatofugal flow of MTs in developing axons, first observed in 1940 using axonal constrictions [364] and later confirmed in vertebrates and flies alike [365–368]. Forces contributing to this process could be derived from an increase in MT volume through polymerisation along the axon shaft [77], pulling forces in the rear of growth cones [246], thermal motion of MT-MT cross-linkers [369], kinesins actively sliding MTs along other MTs ('B' in Fig. 3; [370]), or dyneins sliding MTs along cortical F-actin ('10' in Fig. 3; [88, 258, 371, 372]).

Potential MT sliding along cortical actin would represent one form of tethering MT bundles to the axonal surface. Such anchorage is also suggested by observed co-drift of the axolemma with the axon core [248, 251, 373]. But anchorage would not have to be static; for example, it might involve an interface of slip-bonds, as similarly suggested for actin networks that flow across, whilst dynamically anchoring to, stable focal adhesion sites [374]. MTs could anchor to cortical actin (Fig. 1; '2' in Fig. 3; [20]) or to membrane-associated or transmembrane proteins including ion channels, ion transporters or adhesion factors (Fig. 1; '3' in Fig. 3). Links to transmembrane proteins could be used as mechano-sensing modules [375] that measure local shear forces generated between MT bundles and the axonal environment (Fig. 1). Such mechano-sensing properties could explain local regulation phenomena: for example, net rates of mitochondrial movement along the axon are fairly constant, but the slow transport component (driven by MT bundle flow) is low in proximal and high in distal axon segments; this gradual increase in the amount of slow transport is compensated for by inverse proportional amounts of fast transport (high proximal, low distal [366]). This well-balanced regional amount of fast mitochondrial transport could potentially be regulated by mechano-sensing, i.e. measuring the local MT drift rate relative to the outer axonal environment in each axon segment.

Apart from dynein (see above), other potential anchoring mechanisms can be deduced from the literature. For example, spectraplakins are good candidates, as suggested by distal shift of axonal MTs in fly neurons lacking the *Drosophila* spectraplakin Shot and treated with the MT-stabilising drug taxol [90]. Three distinct mechanisms could account for spectraplakin-mediated MT anchorage: Firstly, spectraplakins could directly cross-link actin and MTs ('2' and '5' in Fig. 3). Secondly, they could link to membrane-associated proteins; for example, the mammalian spectraplakin dystonin can link to ß4-integrin and transmembrane collagen XVII ('3' in Fig. 3; [90]), and *Drosophila* Shot is able to regulate the axonal localisation of the cell adhesion molecule Fasciclin 2, potentially cross-linking Fasciclin 2 to MT bundles [376, 377]. Thirdly, spectraplakins were shown, in non-neuronal cells of fly and mammals, to anchor MT minus ends to the cortex ('1' in Fig. 3; [225, 378, 379]); this mechanism requires interaction with the MT minus end-stabilising factor CAMSAP/Patronin, a factor that is known to be relevant for neuronal morphology [380].

Also other MT-binding proteins, such as tau, MAP1B, APC and dynamin, might be involved in anchorage since they were also reported to bind to actin or to the cortex ('2' in Fig. 3; [324, 344, 346, 347, 381–384]). Potential MT-actin cross-linkage in the axon may not only occur at the cortex, but as well at central longitudinal actin trails (Fig. 1; [17, 24]), thus further contributing to the intricate cross-linking networks expected to stabilise MT bundles. Deciphering MT bundle cross-linkage, internally or with the axonal surface, stays a major challenge for future research, but its understanding will teach us important lessons about axon biology and pathology.

## Conclusions and future perspectives

Here we have presented a conceptual view by describing our vision of a functional interactome of cross-regulatory networks acting at the local level in axons. This model sits right at the interface of research into molecular mechanisms and modern bioinformatics approaches of functional network analysis. We propose that there has to be a fine balance between damaging effects inflicted by life-sustaining motor movements ('associated', 'A-E' in Fig. 3) and those factors that maintain the highways required for this movement (MT-'taming' mechanisms; '1-17' in Fig. 3); both are fine-tuned through a number of cross-regulatory mechanisms ('a-e' in Fig. 3).

Our model integrates a broad range of findings from the literature. But its original foundations are derived from our own work in *Drosophila* neurons, as a consistent cellular system in which it is feasible to study a wide range of genetic factors in parallel and in combination, as a means to understand redundancies, hierarchies and cooperation [46, 70, 72]. This offers unique possibilities to tackle the daunting task of disentangling the enormous complexity of axonal MT bundle regulation. For this, the model of local axon homeostasis could provide a useful basis, helping to develop testable working hypotheses; a good starting point might be to break down the local axon homeostasis machinery into classifiable sub-machineries, like those discussed in the different sections of this review.

This approach also means that we need to recognise the value of incremental long-term approaches that gradually assemble known and newly discovered molecular mechanisms into an integrated understanding of how axon biology is orchestrated at the cellular level, i.e. the organisation level at which axonopathies become manifest. As B.A. Cohen put it: "Research that results in models that reliably and quantitatively predict the outcomes of genetic, biochemical, or pharmacological perturbations should be valued highly, and rewarded, regardless of whether such models invoke novel phenomena" [385].

For the studies of MTs in neurons, we need to take into consideration that knowledge derived from non-neuronal cells might only partly apply [72]. Furthermore, the interactome shown in Fig. 3 makes clear that we will need quantitative approaches: we know increasingly well how factors bind to MTs and partly understand how they might compete with each other. But how crowded can a single MT be, how many molecules are there in its surrounding at any time point, and how much dynamic exchange is taking place? Computational modelling will be an unavoidable means to make sense of existing data and make reasonable predictions to inform experimentation [386, 387].

Integrated understanding of axon biology will also improve our knowledge of the next higher level of complexity, i.e. the signalling networks and systemic factors (e.g. second messengers, ATP, ROS, the 'tubulin code' [104, 113, 212, 388]) that govern axon homeostasis and that maintain balance even during phases of change (e.g. when switching from growth to differentiation, or during stress, injury, regeneration) - or that tip the balance and induce degeneration in disease or ageing. Also the roles of glial cells, likely acting as important external influencers of such systemic processes [389], will become clearer.

Finally, MTs have been recognised as promising therapeutic targets [302, 390, 391], and urgently needed advance on this translational path will be facilitated by a better understanding of the axonal MT homeostasis system. A larger focus of the research community on MTs and, in turn, an improved availability of MT-related data that can be incorporated into our understanding, would be a key prerequisite to make such progress.

## Data Availability

Data sharing not applicable to this article as no datasets were generated or analysed during the current study.

## Abbreviations

AAA: ATPases associated with diverse cellular activities
ACF7: Actin cross-linking factor 7
APC: Adenomatous polyposis coli
CAMSAP: Calmodulin-regulated spectrin-associated protein
CFEOM1: type 1 congenital fibrosis of the extraocular muscles
Eb1: End binding protein 1
Efa6: Exchange factor for ARF6
ɣTuRC: ɣ-tubulin ring complex
HSAN6: type 6 hereditary sensory and autonomic neuropathy
iPSC: induced pluripotent stem cell
Khc: Kinesin heavy chain
KIF: Kinesin family member
KLC: Kinesin light chain
MIP: Microtubule inner proteins
MT: Microtubule
MTED: Microtubule elimination domain
MTOC: MT organising centres
PF: Protofilament
PRR: Plakin repeat region
ROS: Reactive oxygen species
Shot: Short stop
SPG4: Spastin-linked spastic paraplegia
STED: Stimulated emission depletion
